# Development and Regeneration in the Endocrine Pancreas

**DOI:** 10.5402/2012/640956

**Published:** 2012-12-27

**Authors:** Ahmed Mansouri

**Affiliations:** ^1^Research Group Molecular Cell Differentiation, Department Molecular Cell Biology, Max-Planck Institute for Biophysical Chemistry, Am Fassberg 11, 37077 Goettingen, Germany; ^2^Department of Clinical Neurophysiology, University of Goettingen, Robert-Koch-Strasse 40, 37075 Goettingen, Germany

## Abstract

The pancreas is composed of two compartments that deliver digestive enzymes and endocrine hormones to control the blood sugar level. The endocrine pancreas consists of functional units organized into cell clusters called islets of Langerhans where insulin-producing cells are found in the core and surrounded by glucagon-, somatostatin-, pancreatic polypeptide-, and ghrelin-producing cells. Diabetes is a devastating disease provoked by the depletion or malfunction of insulin-producing beta-cells in the endocrine pancreas. The side effects of diabetes are multiple, including cardiovascular, neuropathological, and kidney diseases. The analyses of transgenic and knockout mice gave major insights into the molecular mechanisms controlling endocrine pancreas genesis. Moreover, the study of animal models of pancreas injury revealed that the pancreas has the propensity to undergo regeneration and opened new avenues to develop novel therapeutic approaches for the treatment of diabetes. Thus, beside self-replication of preexisting insulin-producing cells, several potential cell sources in the adult pancreas were suggested to contribute to beta-cell regeneration, including acinar, intraislet, and duct epithelia. However, regeneration in the adult endocrine pancreas is still under controversial debate.

## 1. Introduction 

The pancreas is an important organ that produces digestive enzymes and hormones to control blood glucose homeostasis. Hence, the organ consists of two major compartments. The main part, the exocrine tissue is composed of acinar cells and an intricate ductal system to transport the digestive juice to the duodenum. Embedded within the exocrine tissues reside highly organized functional units called islets of Langerhans where five hormone secreting cells are clustered [1–5]. In the mouse, islets typically display a core of insulin-producing beta-cells surrounded by alpha, delta, PP, and epsilon cells, secreting the hormones glucagon, somatostatin, pancreatic polypeptide, and ghrelin, respectively [1–5]. 

During mouse pancreas genesis a complex and highly orchestrated molecular program acts to control the allocation of cell progenitors towards mature endocrine cells [1, 6, 7]. The delineation of the pancreatic fate is marked by the coexpression domain of the transcription factors *Pdx1* and *Ptf1a* at the foregut/midgut junction, where a ventral and a dorsal evaginations announce the first morphological sign of pancreas development [6, 8–10]. Lineage tracing experiments clearly demonstrated that *Pdx1*-positive progenitor cells contribute to the formation of endocrine and exocrine compartment [11]. Similarly, all *Ptf1a*-positive cells generate all pancreatic derivatives [10]. In the absence of *Ptf1a* gene activity pancreatic cells destined to form the ventral pancreas adopt a duodenal epithelium phenotype, indicating that *Ptf1a* is required to confer endodermal progenitors with pancreatic fate by repressing the allocation to intestinal destiny [10]. The pancreatic epithelium undergoes growth, branching, and differentiation with the onset of the so-called secondary transition at embryonic day 13.5 (E13.5) of gestation [12]. At E12.5 fusion of ventral and dorsal pancreas occurs. Genetic lineage tracing experiments demonstrated that multipotent progenitor cells reside at the tip of the branching epithelium and are marked by the expression of *Pdx1*, *Ptf1a*, Cpa1, and c-myc [13]. These multipotent progenitors differentiate into acinar, duct, and endocrine cells to eventually become restricted to exocrine fate after E14.0 of development [5, 13]. 

A highly debated issue is whether endocrine progenitors exist in the adult pancreas. Several animal models of pancreas injury have been analyzed for their capacity to undergo regeneration [14–22]. In these animals endocrine cell regeneration was reported. Thus, various sources of islet neogenesis were proposed. It appears that one major and dominant mechanism to regenerate insulin-producing cells, under injured and physiological conditions, consists in the capacity of preexisting beta-cells to self-replicate [23–25]. In addition, intraislet progenitors as well as centroacinar cells have been suggested as a site of islet neogenesis [18, 19, 26, 27]. Moreover, several studies indicate that in the adult pancreas duct and/or duct-lining cells have the propensity to differentiate into endocrine cells and give rise to functional beta-cells [16, 28–33]. Of high interest is the observation that compromising the glucagon-signaling pathway is associated with alpha-cell regeneration [22]. Analyses of knockout mice lacking factors implicated in pancreas development have made the path for the establishment of protocols to generate insulin-producing cells from progenitor and/or embryonic stem cells [34].

## 2. Factors Controlling Pancreatic Endocrine Progenitors

The first signs of pancreas development appear as a dorsal and a ventral bulge at the foregut/midgut junction. Although at E9.5 few cells in the pancreatic epithelium start to express glucagon, and, 24 hours later some of these coexpress insulin, they do not appear to contribute to the mature endocrine pancreas [40]. However, glucagon has been shown to affect the differentiation of early insulin-expressing cells in the developing mouse pancreas [41, 42]. It is first during the secondary transition at E13.5 where the amplification of endocrine cells takes place [12]. The expression of *Fgf10* in the pancreatic mesenchyme is required for the proliferation of the pancreatic epithelium, where growth and branching in *Fgf10*-deficient mice are dramatically affected [43]. In addition, the gene activity of *Isl-1* is necessary for the formation of the dorsal mesenchyme. Accordingly, in *Isl-1 *mutant mice the dorsal mesenchyme is not formed, and islet cell genesis is affected [44]. Thus, it is important to notice that Isl-1 is necessary in the mesenchyme, as well as in the pancreatic epithelium for normal pancreas development [44]. 

All pancreatic endocrine progenitors are marked by the expression of the bHLH transcription factor *Ngn3* [45, 46]. In most organs Notch signaling was found to control cell fate decisions. This is also true for the pancreas, as documented by the expansion of endocrine progenitors in mice where Notch signaling is compromised. Accordingly, the loss of gene activity in Notch signaling components such as DLL1, RBPJ-*κ*, or *Hes1* is accompanied by a dramatic increase in the content of *Ngn3*-labeled cells [47, 48]. Moreover, the Notch downstream target *Hes1* was shown to bind to the proximal *Ngn3* promoter and inhibit transcription [49]. On the other hand, sustained Notch signaling pathway in pancreatic progenitors where the expression of activated Notch is induced under the control of *Pdx1* promoter prevents endocrine as well as exocrine differentiation [50]. Hence, in a recent study using genetic lineage tracing, *Hes1*-labeled cells were found to contribute to exocrine and endocrine cells in the developing pancreas [51]. Interestingly, Notch signaling not only activates *Hes1*, a repressor of *Ngn3*, but also promotes the expression of *Sox9*, an activator of *Ngn3* [52]. This discrepancy is concealed by the recent finding demonstrating different level of Notch activity required to induce Sox9 and *Hes1* expression in the pancreas and thereby controls the segregation of cellular fates [52]. On the other hand it is interesting to notice that presenilins dose was also found to regulate the fate of pancreatic endocrine progenitors. *Ngn3*-positive cells where presenilin was inactivated became fated to acinar cells [53]. Thus, during a narrow window of endocrine development Notch2 was identified as a crucial player, sustaining the selection of endocrine cell destiny by *Ngn3*, where it contributes to titrating RBP-J*κ* from *Ptf1a* [53]. 

Beside Notch also TGF-*β* signaling appears to act on progenitor cells in the pancreas and GDF11 as well as Smad2 were found to affect pancreatic endocrine cell differentiation. In fact, in the absence of *Gdf11* or *Smad2* gene activity, an expansion of *Ngn3*-labeled cells was uncovered [54]. Thus, the expression of *Hes1* in the pancreas of *Gdf11* or *Smad2 *mutant mice was not altered, and suggesting that Notch and TGF-*β* signaling may act in parallel pathways to control pancreatic endocrine cell progenitor expansion [54].

During mouse development pancreatic endocrine cell genesis is initiated by the activation of the bHLH transcription factor *Ngn3* in the pancreatic epithelium. *Ngn3* labels all endocrine progenitors [45, 46, 55, 56]. Accordingly, *Ngn3* was found necessary for the formation of the endocrine pancreas, and its forced expression under the control of the *Pdx1* promoter is sufficient to promote the generation of all endocrine cells [46, 55]. Interestingly, the early activation of *Ngn3* led to the production of mostly glucagon-labeled cells, while the induction of *Ngn3* at later stages of development promotes the formation of insulin, somatostatin, and PP cells [55]. However, by which molecular mechanism *Ngn3* mediates endocrine fate choice is still unclear. For instance, the manipulation of *Ngn3* expression level in endocrine progenitors uncovered the critical role played by *Ngn3* expression threshold to promote an endocrine cell destiny [57]. It appears that high levels of *Ngn3* protein are necessary to engage pancreatic cell progenitors into the endocrine fate while pancreatic progenitors exhibiting low levels of *Ngn3* give rise to acinar or duct cells. Thus, high level of *Ngn3* expression was suggested to possibly act in feedback loops involving lateral inhibition mechanism [57]. Moreover, *Ngn3* was shown to be involved in the initiation of the delamination of endocrine cells from the pancreatic epithelium, whereby effectors of epithelial mesenchymal transition (EMT) are activated to repress the expression of E-cadherin [58]. This is corroborated by a recent study showing that *Ngn3*-labeled progenitors play a role in controlling fate and morphogenesis of the pancreatic duct epithelium [59]. The role of *Ngn3* as a determinant of endocrine progenitors is further documented by its ability to activate the transcription of a number of genes acting in the process of differentiation and subtype specification of various pancreatic endocrine hormones. Such factors include Pax4, Nkx2.2, NueroD1, and the direct downstream target of *Ngn3* insulinoma-associated 1 (Insm1) [3, 60–63]. Remarkably, *in vivo* clonal analysis in mice elegantly provided strong evidence that *Ngn3* expressing cells are unipotent and low replicating cells [64]. This may have implications for the generation of insulin-producing cells from progenitor cells or embryonic stem cells. 

Several other transcription factors control upstream of *Ngn3* the development of the endocrine pancreas. Thus, the transcription factor Sox9 was found to label pancreatic multipotent progenitors, but its expression is downregulated in committed endocrine progenitors and differentiated cells [65]. In adult pancreas Sox9 is restricted to duct cells, and its loss uncovered a role upstream of *Ngn3* in promoting progenitor cell proliferation, and survival [65]. Moreover, Sox9 was also suggested to mark those pancreatic epithelial cords in the developing pancreas that differentiate into exocrine and endocrine cells [66]. 

The transcription factor Nkx6.1 is expressed in pancreatic epithelium and is later confined to the developing beta-cell [67]. In mice lacking Nkx6.1 differentiating beta-cells are not formed, indicating that insulin-producing cell depletion occurs by apoptosis. Interestingly, Nkx2.2/Nkx6.1 compound mutants exhibit a similar phenotype to Nkx2.2, suggesting a role for Nkx2.2 upstream of Nkx6.1 [67]. Recent gain-of-function experiments where Nkx6.1 forced expression was induced in the endocrine pancreas revealed a notch-dependent and cross-inhibitory interaction mechanism operating between Nkx6.1 and *Ptf1a*, to endow progenitor cells with ductal/endocrine or acinar cell destiny, respectively [68]. 

Genetic analysis has demonstrated that the transcription factors HNF1*β* and HNF6 label early pancreatic progenitors, act upstream of *Ngn3*, and are thus required for the proper differentiation of *Ngn3*-expressing cells [69, 70]. Lineage tracing experiments using the Cre recombinase under the control of the HNF1*β* promoter provide clear evidence that HNF1*β* expression undergoes a developmentally regulated restriction [71]. First, HNF1*β* is found in pancreatic progenitors, subsequently becomes confined to those epithelial cells (termed also embryonic cord) that will give rise to ductal and endocrine cells, to eventually persist only in adult duct cells [71]. In summary, while *Ngn3* marks endocrine progenitors, pancreatic epithelium expressing Sox9, Hnf1*β*, or Cpa1 appear to label multipotent progenitors giving rise to all pancreatic cells and including endocrine lineage [4, 5, 13, 22, 72]. Moreover, the further restriction of multipotent pancreatic progenitor to ductal and endocrine fate is marked by the mutual expression of Sox9 and HNF1*β* in embryonic cord cells [66, 71]. Thus, the allocation to different cell fates is clearly endowed by the differential expression of transcription factors.

Finally, in contrast to previously assumed, *Ngn3* expression has been found sustained in adult islets, and genetic analysis provided evidence for a role for this factor in contributing to islet maturation and preserving islet function [73].

## 3. Endocrine Cell Subtype Specification

Following *Ngn3* activation a battery of transcription factors is induced to promote the allocation of endocrine progenitors to distinct hormones producing cells [1–3, 7]. Most of these factors appear at early stages of development in the pancreatic epithelium and progressively exhibit a more restricted expression pattern with advanced endocrine cell genesis. The first induced factors during the initiation of pancreatic endocrine differentiation program, and downstream of *Ngn3*, are NeuroD1 (BEATA2), Insm1, and Rfx6 which appear as immediate targets of *Ngn3* [63, 74–77]. Accordingly, the loss of Insm1 or Rfx6 is accompanied by the persistence of mostly nondifferentiated islet progenitors. Interestingly, similar to *Ngn3*, the forced expression of NeuroD1 in pancreatic progenitors under the control of the *Pdx1* promoter results into differentiation to hormone producing cells, while the of loss function provokes a of loss insulin producing beta-cells by apoptosis [46, 74].

Subsequently, endocrine progenitors are allocated to the different hormone producing cells. The molecular mechanism underlying endocrine cell subtype specification is still not completely understood. However, the analysis of gain- and loss-of-function mutant mice revealed a complex cross-regulatory interaction between transcription factors and involving very often reciprocal inhibitory mode of operation. This is illustrated for two transcription factors, Pax4 and Arx, playing a crucial role in the specification of insulin and glucagon cell destiny, respectively [1, 2, 7]. Pax4, a paired box-containing factor, is first detected in the pancreatic epithelium and later restricted to the beta/delta-cell lineage [78, 79]. A further study using lineage tracing indicates that Pax4-positive cells represent specified endocrine progenitors that may contribute equally to islet cells [80]. Arx is also found in the pancreatic epithelium and later confined to alpha and PP cells [81]. Pax4-deficient pancreas exhibit normal islet morphology but is devoid of insulin-producing beta-cells and somatostatin-labeled delta-cells. Instead, a proportionally increased number of glucagon expressing cells is accumulating in the core of the islet [78]. In contrast, in *Arx-*deficient animals alpha-cells do not develop, whereas an augmentation of the beta- and delta-cell content is detected [81]. It appears that *Pax4* and *Arx* mutant mice suffer from opposite phenotypes in the endocrine pancreas, clearly suggesting that these two factors interact with each other. These findings sustain a working model where Pax4 and Arx are first expressed in the same proendocrine cell and undergo a reciprocal inhibitory interaction to endow endocrine progenitors with a beta-/delta-cell fate or alpha-cell destiny, respectively. This is corroborated by the ability of the Pax4 factor to bind Arx promoter sequences and repress *Arx* transcription, and similarly Arx protein to suppress *Pax4* transcription by binding to *Pax4* promoter [82]. The signal that triggers the segregation of Pax4- and Arx-expressing cells is not known. However, the repression of Arx in committed beta-cells is mediated by methylation of *Arx* locus [83]. Arx is thus hypomethylated in alpha-cells, as well as in *Dnmt1*-deficient beta-cells that undergo conversion to alpha-cell destiny [83]. It is also worth mentioning that Isl-1 was recently found to regulate *Arx *transcription in alpha-cells [84].

The molecular characterization of pancreata, derived from mice lacking Pax4 and/or Arx, has provided further insights into the molecular mechanisms controlling pancreatic endocrine cell subtype specification and demonstrated that, in a second round of endocrine cell allocation, a similar interaction may occur between Pax4 and a factor X which may contribute to the promotion of beta- and delta-cell destiny, respectively. Gain-of-function experiments where Arx was misexpressed in adult beta-cells provided further insight that Arx is also sufficient to force the alpha- and PP-cell fate even in mature beta-cells [85]. Along the same line of evidence, the sole forced expression of Pax4 in glucagon-producing cells is able to reprogram alpha-cells into functional beta-cells that can counter chemically induced diabetes. Remarkably, the forced expression of Pax4 also triggered an *Ngn3*-dependent and duct-derived cycle of alpha-cell regeneration (see later).

Beside Pax4 and Arx, Nkx2.2 was found to play an important role in endocrine cell differentiation. Thus, Nkx2.2 is detected at E9.5 in the dorsal pancreatic epithelium and later confined to beta-, alpha-, and PP-cells [86]. Hence, in the absence of this factor, alpha- and beta-cell development is severely affected, while the number of ghrelin-positive cells is increased. Instead, immature beta-cells are detected in Nkx2.2^−/−^ pancreas, suggesting an important function for Nkx2.2 in beta-cell differentiation [86]. Interestingly, the transcription of Arx was found enhanced in the pancreata of these mutants [87–89]. Analysis of pancreata derived from mice lacking functional Arx and Nkx2.2 support the idea that Nkx2.2 acts to reinforce the transcriptional networks initiated by Pax4 and Arx in early committed beta- and alpha-cells, respectively. Moreover, it appears that the coupled function of Pax4 and Nkx2.2 is to counteract Arx gene activity in early committed beta-cells [88, 89]. Remarkably, Nkx2.2 was found engaged in a repressor complex including DNMT3, Grg3, and HDAC1, to promote beta-cell differentiation, and thereby inhibiting alpha-cell destiny [90].

The predominant role of Pax4 and Nkx2.2 in beta-cell differentiation is further documented by a study showing that in the absence of Pax4, Nkx2.2, or both factors, the expression of beta-cell markers including insulin, *Pdx1*, HB9, and Nkx6.1 is abolished. Moreover, the two factors act in parallel to promote beta-cell differentiation program [79]. 

Several transcription factors such as *MafA*, MafB, *Pax6*, Pax4, Nkx6.1, *Pdx1*, and Isl-1 are still expressed in the adult pancreas and appear to sustain endocrine cell fate. *MafA* and MafB factors display a developmentally highly regulated expression pattern that correlates with beta- and alpha-cell maturation, respectively [91]. Accordingly, MafB expression starts in the pancreatic epithelium at E10.5 and is found in developing beta- and alpha-cells to eventually become confined to glucagon-expressing cells after partum [92–94]. In contrast, *MafA*-labeled cells first appear at E13.5 in insulin-positive cells, and *MafA* expression persists in adult beta-cells [92, 95]. The dominant role of *MafA* in beta-cell maturation is documented by the regulation of its activity by several pancreatic transcription factors and including Nkx2.2, Nkx6.1, NeurD1, Foxa2, *Pdx1*, *Pax6*, MafB, and Isl-1 [91]. Moreover, the forced expression of *MafA* together with *Ngn3* and *Pdx1* is able to convert acinar cells into functional beta-cells *in vivo* [35].

*Pax6* is already detected in the pancreatic epithelium at E10.5, and its absence provokes a decrease of all islets cells, with the exception of ghrelin-labeled cells [96–98]. However, the conditional inactivation of *Pax6* in the endocrine pancreas does not affect endocrine cell genesis and reveals that this factor is required for maintaining cell differentiation [99]. Accordingly, the beta-cell determinant factor Nkx2.2 is necessary to sustain the proper expression level of *Pax6* in beta-cells [79]. Nkx6.1 expression in adult beta-cells was shown to stimulate their proliferation *in vitro* [100]. However, the forced expression of this factor in adult beta-cells of transgenic mice has no impact on beta-cell proliferation or regeneration [101]. Pax4 function in adult beta-cells is still unclear, notwithstanding Pax4 was shown to play a role *in vitro* in the proliferation and survival of insulin-producing cells [102]. Finally, Isl-1 is required in the pancreatic mesenchyme and epithelium to promote pancreas development [44]. Recent study revealed the role of Isl-1 in the endocrine pancreas where it affects proliferation and survival of islet cells. Moreover, *MafA* a beta-cell maturation determinant was identified as a direct target of Isl-1 [103]. 

While the expression of *Pdx1* labels all pancreatic progenitors, it eventually becomes confined to the developing beta-cell where it persists in the adult pancreas. Accordingly, the global loss of *Pdx1* is accompanied by the development of a rudimentary pancreatic bud [8, 9], while the conditional inactivation reveals a role for this factor in sustaining the beta-cell phenotype through the suppression of glucagon gene activity [104]. Remarkably, the forced expression of *Pdx1* in endocrine progenitors is able to convert alpha- into beta-cells, whereas alpha-cells are resistant to beta-cell reprogramming through *Pdx1* activity [105]. It is interesting to remind that, in contrast to *Pdx1*, the sole expression of Pax4 is sufficient to reprogram alpha-cells into insulin-producing beta-cells that counter chemically induced diabetes [32].

## 4. Endocrine Cell Regeneration in the Adult Pancreas 

**Figure 1:**
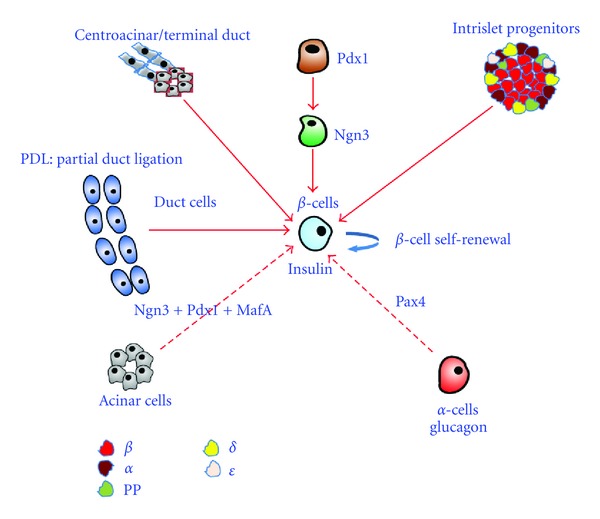
Regeneration routes in the adult endocrine pancreas. During normal development *Pdx1*-marked cells are fated to generate pancreas tissue. Subsequently, a small proportion of these cells labeled by the bHLH factor *Ngn3* acquire endocrine cell destiny and give rise to hormone-producing cells, and consisting of beta-, alpha-, delta-, PP-, and epsilon-cells, producing insulin, glucagon, somatostatin, pancreatic polypeptide, and ghrelin, respectively. Following pancreatic injury different sources of stem/progenitor cell were proposed. It is well accepted that beta-cells are able to self-renew under physiological conditions or following pancreatic injury. However, other sources of beta-cell regeneration are under debate. In several animal models duct/duct-lining cells appear as the dominant source where stem/progenitor cells may reside. Interestingly, the forced expression of *Pdx1*, *Ngn3,* and *MafA* is able to allow transdifferentiation of acinar cells into insulin-producing cells *in vivo* [35]. Global ablation of beta-cells using diphtheria toxin led to some conversion of alpha- to beta-cells [20]. A more robust transdifferentiation of alpha- to beta-cells was observed following the combination of pancreatic duct ligation and alloxan-induced beta-cell injury [36, 37]. On the other hand, the misexpression of a single factor, Pax4 in alpha-cells, was able to endow these with functional beta-cell characteristic, and counter chemically induced diabetes in mice. This study clearly uncovered the robust-regenerative capacity of alpha-cells and provides a possible new source for generating beta-cells to develop revolutionary approaches to treat diabetes [32, 38, 39]. PDL: pancreatic duct ligation.

Understanding the molecular mechanisms controlling endocrine pancreas development, those operating during islet neogenesis in pancreatic injury models may open new avenues to develop novel approaches for the treatment of diabetes. Several independent studies using various models of pancreatic injury revealed that regeneration may occur in the adult pancreas [22, 106, 107]. Thus, different models and mechanisms of endocrine cell regeneration were proposed. Moreover, the mechanisms underlying endocrine cell neogenesis appear to depend not only on the type but also on the extent of injury [22]. Therefore, it is still controversially debated whether stem/progenitor cells exist in the adult pancreas, and if so where do these may reside (Figure 1). 

The major mechanism leading to regeneration of insulin-producing cells in the adult pancreas is self-renewal of preexisting beta-cells. However, in several studies also intraislet progenitors were proposed to contribute to islet neogenesis [18, 19, 26, 36]. Although lineage tracing experiments have provided strong evidence that preexisting acinar cells do not contribute to endocrine cells [108], acinar AR42J cells were shown to give rise to insulin- and glucagon-producing cells, when treated with betacellulin, activin, or glucagon-like peptide [109, 110] (for review see [22]). In addition, centroacinar/terminal duct cells were found to express stem cell markers and may therefore represent a source of progenitor cells engaged in islet regeneration [29, 111]. The transdifferentiation of acinar cells into functional beta-cells in mice can be induced by the ectopic combined expression of three factors, *Pdx1*, *Ngn3*, and *MafA*, and clearly document the inherent capacity of the acinar compartment to give rise to islet cells [35]. This is corroborated by a recent study in zebrafish, demonstrating that suppressing *Ptf1a* gene activity in the acinar compartment of postembryonic pancreas is able to promote transdifferentiation into endocrine cells [112]. In addition, in several studies of pancreatic injury models, and transgenic mice, progenitor/stem cells were suggested to reside in the duct epithelium, where cells expressing the proendocrine marker *Ngn3* were detected. Of interest is further the demonstration of the robust regeneration capacity of alpha-cells, following alterations in the glucagon signaling pathway [22].

## 5. Self-Replication of Preexisting Beta-Cells 

It is now well accepted that preexisting insulin-producing cells have the ability to undergo self-renewal under normal physiological condition [23, 113] and following injury. Thus, genetic as well as DNA analog-based lineage tracing experiments have provided strong evidence for the propensity of the beta-cell to self-replicate in different injury models, including pancreatectomy, diphtheria-toxin-induced beta-cell ablation, and pancreatic duct ligation [17, 23–25]. This is also true for beta-cell replenishment occurring in animals where c-myc conditional overexpression induced beta-cell apoptosis, and leading to diabetes. Following inactivation of c-myc overexpression insulin-producing cells regenerate and diabetes is reversed [114]. These studies are further sustained by the finding that, following partial pancreatectomy in mice, beta-cell regeneration occurs in the absence of the proendocrine marker *Ngn3* reactivation [115]. This is in agreement with a report showing that miRNAs accumulate in the adult pancreas subjected to pancreatectomy, and some of these were identified as candidates with the capacity to block *Ngn3* expression [116]. Furthermore, it appears that the increase of beta-cell mass in obese individuals and during pregnancy is driven by the amplification of preexisting beta-cells [117]. Of interest is the observation that the endocrine tumor suppressor Menin is downregulated in the islets during pregnancy and leading to the growth of beta-cell mass [118]. In addition, STAT5, growth hormones, prolactin, and foxM1 were shown to promote beta-cell self-renewal during pregnancy [117]. However, non-beta-cell progenitors were also reported to contribute to the increase in beta-cell mass during pregnancy in mice [119]. Furthermore, it has also to be mentioned that, following streptozotocin-mediated islet injury, and during aging in mice, a lineage-tracing study using a similar approach as described by Dor et al. [23] provided evidence that, beside beta-cell self-renewal, intraislet progenitors may participate in beta-cell regeneration [18].

Beta-cell proliferation is age dependent, for instance, in rodents, and regeneration capacity is therefore dramatically reduced in older mice, as compared to young animals [120–123]. Of note is the role of cell-cycle regulators in controlling beta-cell replication, documented by the reduced islet size, and beta-cell replication potential found in the pancreas of cyclin D1, 2-deficient mice [124, 125]. Beta-cell proliferation may also in human contribute to the increase in beta-cell mass, as implicated from *in vitro* experiments where human islets were forced to express the Cdk4 gene [126].

## 6. Facultative Stem Cells May Reside in the Duct Epithelium

 In addition to beta-cell self-renewal other mechanisms may act to promote endocrine cell regeneration in the adult pancreas. Thus, several sources of progenitor/stem cells in the adult pancreas were proposed to contribute to islet cell neogenesis and give rise to functional beta-cells. The most prominent model being the delamination of endocrine cells is residing in the duct epithelium, where an *Ngn3*-dependent endocrine cell neogenesis is initiated. Using several models of pancreas injury, and including transgenic animals, endocrine cells expressing insulin, glucagon, but also *Ngn3*, as well as *Pdx1* labeled cells were detected in the ductal epithelium, providing evidence that indeed ductal lining structures may comprise multipotent facultative stem/progenitor cells [22, 107, 127, 128]. In fact, partial duct ligation (PDL) in the pancreas was accompanied by the emergence of *Ngn3*-labeled duct cells that are able to differentiate in all endocrine cells [30]. This is corroborated by a study using carbonic anhydrase II promoter to genetically mark duct derivatives in PDL injury model and demonstrating that duct cells have indeed the capacity to give rise to endocrine as well as exocrine cells [31]. The combination of PDL with the ablation of beta-cells through alloxan also resulted in robust beta-cell regeneration. However, the newly formed beta-cells do not appear to derive from the duct epithelium but through direct conversion of alpha-cells [36]. Hence, beta-cell neogenesis was suggested to occur from intraislet progenitors [36]. On the other hand, another mouse model, where alpha-cells were directly converted into functional beta-cells by the conditional misexpression of the transcription factor Pax4 in glucagon-producing cells, resulted in glucagon neogenesis through the activation of duct-derived facultative stem cells [32, 38]. This clearly illustrates the impact of the type and extent of pancreatic injury under study on the regeneration process. Of note is the observation that beta-cell neogenesis also occurs in BETA2/NeuroD1 knockout mice that survived to adulthood. Newly generated beta-cells in these animals were found to derive from two distinct sources, by self renewal of preexisting beta-cells, and from the duct epithelium [129]. 

Lineage tracing experiments using Hnf1*β* driven Cre recombinase to label duct progenitors in PDL- or alloxan/EGF/gastrin-injured adult pancreas revealed no contribution of duct epithelium to endocrine regeneration [71, 130]. Similarly, in the adult non-injured pancreas Muc1 lineage-labeled cells were only found to participate in the formation of duct/acinar cells [131]. Moreover, also Sox9 lineage-labeled cells in various pancreatic injury models did not uncover any endocrine cells derived from duct epithelium [132]. This contrasts the findings where Carbonic anhydrase II was used for genetic lineage tracing, and may suggest that Hnf1*β*Cre, Sox9Cre, as well as Muc1Cre do not really mark all duct cells [31]. It is therefore reasonable to assume that progenitor/stem cells may consist of a rare population and/or reside near duct structures (duct lining). Moreover, these discrepancies clearly suggest that there is a need for additional pancreatic injury models as well for transgenic mouse lines allowing lineage tracing of duct and/or acinar cells [133]. The finding that duct cells may contain facultative stem cells [128] implicates that cells has to delaminate from these epithelial structures and migrate to populate the islets of Langerhans.

## 7. Glucagon for Use as Potential Stem Cell of Endocrine Cell Regeneration

Several studies in mice clearly provide strong evidence that defective or altered glucagon-signaling pathway ultimately triggers regeneration of glucagon-producing alpha-cells. This has been shown in several animal models where glucagon shortage occurs. As mentioned above the forced expression of Pax4 in glucagon-expressing alpha-cells is able to convert these into functional beta-cells that counter chemically induced diabetes in mice [32, 38]. Transgenic mice ectopically expressing Pax4 in glucagon-producing cells develop an age-dependent increase in islet size and beta-cell mass. Remarkably, the so induced transdifferentiation of alpha-cells revealed that, in order to compensate the thereby compromised glucagon signaling, alpha-cell neogenesis occurred in these mice. As a consequence a permanent cycle of alpha-cell/regeneration and conversion into beta-cell, was suggested, eventually contributing to increased islet size [32, 38]. The observed alpha-cell renewal in Pax4-transgenic mice is corroborated by glucagon hyperplasia observed in mice lacking glucagon receptor, prohormone convertase 2, or glucagon gene-derived peptides [134–136]. Moreover, glucagon supplementation is able to affect the phenotypes of oversized islets and/or glucagon hyperplasia observed in these mice [32, 137]. These studies clearly establish that deregulated glucagon signaling results in alpha-cell neogenesis, and providing clear evidence that endocrine cell regeneration can be induced in the adult pancreas. Interestingly, mice lacking prohormone convertase 2 gene activity, beside glucagon hyperplasia, also islet neogenesis was reported [138]. 

Using diphtheria toxin to globally ablate glucagon cells in the adult pancreas does not provoke glucagon neogenesis [139]. It appears that as few as two percent of alpha-cell mass cells are sufficient to produce enough glucagon so that glucagon signaling remains unaffected [139]. Notwithstanding, in several animals models described above, glucagon shortage was compensated by alpha-cell amplification. However, it is not clear which signal(s) is triggering alpha-cell neogenesis. It is interesting to notice that the loss of *Menin* gene activity in alpha-cells provokes their subsequent conversion into insulin-producing beta-cells leading to insulinoma formation [140]. In these animals glucagon-producing cells were shown to undergo cell proliferation [140]. Remarkably, spontaneous transdifferentiation of alpha-cells into beta-cells was reported to occur in mice with global ablation of insulin-producing beta-cells by diphtheria toxin [20]. Why such alpha-cell transdifferentiation does not appear to contribute to beta-cell replenishment in diabetic animals remains elusive. It is possible that a signal is required to induce such transdifferentiation mechanism. Glucagon itself may represent such a signal, since as stated above, glucagon supplementation affects the observed alterations of alpha-cell hyperplasia [32, 137]. Remarkably, as reviewed by Liu and Habener, alpha-cell hyperplasia is observed in animals lacking insulin-producing cells such as in Pax4-deficient mice, or where beta-cells were subjected to injury [141]. 

In mice forced to express Pax4 in alpha-cells, duct epithelium was identified as the source of newly generated glucagon-producing cells in an *Ngn3*-dependent manner [32]. Moreover, in glucagon receptor knockout mice glucagon-labeled cells were detected in the duct epithelium [134], and the expression of the proendocrine marker, *Ngn3*, was reactivated in the adult pancreas of these mice [32]. In animals lacking Prohormone convertase 2 gene activity increased alpha-cell proliferation and islet neogenesis were reported [138]. Therefore, it is possible that alpha-cell regeneration as well as alpha-cell self-replication may occur in these animals suffering from alterations in glucagon-signaling pathway. Interestingly, using db/db mice as a model for type II diabetes, it has been recently reported that insulin and glucagon may regulate alpha-cell proliferation [142]. It is important here to recall that, also in other pancreatic injury models such as duct ligation, and streptozotocin-induced beta-cell depletion, stem/progenitor cells were found to reside in the duct epithelium [30]. 

Although some genetic lineage tracing studies in normal and injured animal models have provided no evidence for the contribution of duct or acinar cells to the regeneration of islet cells in the adult pancreas, the alterations observed in mice, where glucagon signaling is affected, clearly indicate that at least glucagon cells are able to regenerate. Remarkably, it has also been reported that in the islets of Type I diabetes patients, an increase in alpha-cell number was detected [143]. Moreover, this is also true for the islets of type I diabetes induced in mice by streptozotocin depletion of beta-cells [144]. All these studies may suggest that glucagon-producing alpha-cells could potentially be used as a source for the generation of insulin-producing cells to replace depleted beta-cells in diabetic patients. Although during normal endocrine pancreas genesis adult insulin- and glucagon-producing cells differentiate from two independent cell lineages, alpha-cells may still constitute a stem cell pool in the adult pancreas. As stated earlier glucagon has been shown to affect the first wave of differentiation of insulin-expressing cells in the developing mouse pancreas [41]. Interestingly, it has been recently proposed that alpha-cells are more plastic and may have the capacity to dedifferentiate to acquire a Pro-alpha-cell state and give rise to beta-cells [145]. This paracrine/autocrine regeneration model suggests that injured beta-cells produce the stromal cell-derived factor 1 (SDF-1) that triggers the dedifferentiation of mature alpha-cells into the Pro-alpha-cell and ensure their proliferation and survival. These Pro-alpha cells are characterized by the expression of the GLP1 and PC1/3 and may acquire mature beta-cell characteristics including the expression of *Pdx1*, *Pax6*, and *MafA* [145]. In order to induce the conversion of alpha- into beta-cells the expression of Pax4 has to be favored at the expense of Arx. Identifying the putative signal(s) that may trigger alpha-cell neogenesis, but also those that normally may act to block alpha-cell conversion into beta-cells in diabetic animals, is therefore of fundamental interest to develop new approaches for the treatment of diabetes.

Finally, although still controversial, most of the studies clearly provide strong evidence for the capacity of the adult endocrine pancreas to undergo regeneration. Several sources of islet cell neogenesis were proposed, and it appears that multiple mechanisms may act during this process. Future studies require novel pancreas injury models to uncover the common dominator of endocrine cell regeneration. Of special interest is the capacity of glucagon cells to regenerate, making alpha-cells as a possible progenitor cell that is prone to transdifferentiation into functional beta-cells.
